# *Trichuris Globulosa* Von Linstow, 1901 from one-humped camel (*Camelus dromedarius*) in Egypt: prevalence, morphological and molecular study

**DOI:** 10.1186/s12917-024-04078-9

**Published:** 2024-06-03

**Authors:** Badawy I. B. Ismail, Mahmoud A. El-Seify, Reda E. Khalafalla, Shimaa S. Sorour, Khaled Sultan, Nagwa M. Elhawary

**Affiliations:** Department of Parasitology, Faculty of Veterinary Medicine, Kafrelsheikh University, Kafr El-Sheikh, 33516 Egypt

**Keywords:** 18s, Camel, Cytb, Egypt, Molecular, *Trichuris Globulosa*, Whipworm

## Abstract

**Background:**

*Trichuris* spp. (whipworms) are soil-transmitted helminths distributed worldwide, parasitizing several mammalian hosts such as ruminants, primates, and rodents. *Trichuris* spp. is one of the most common intestinal parasites affecting both humans and animals, and it can spread directly through the fecal-oral route, resulting in severe illness and financial loss. So, this work aims to detect the frequency of *Trichuris* spp. in camels in Beheira Governorate, Egypt, and to identify *Trichuris* spp. through morphometrical studies, molecular analysis, and phylogenetic analysis.

**Results:**

A total of 35 dromedaries out of 127 investigated had *Trichuris spp*. infection, meaning that the overall prevalence was 27.56%. The age of the camel affected the infection rate, older animals (> 5 years) having a higher prevalence of infection (24%) than animals of ages (< 3 years) (20%) than animals of ages (3–5 years) (19.14%). According to season: *Trichuris* spp. showed a unique pattern in camels in different seasons: summer (31.25%) > autumn (28.13%) > spring (25.8%) > winter (25%) indicating year-round infection. *T. globulosa* was identified morphometrically from camels in Beheira Governorate, Egypt. The BLAST analysis revealed the presence of *T. globulosa* isolate from camels using the Genbank database depending on nuclear small subunit ribosomal RNA (18s) and cytochrome b (Cytb) genes.

**Conclusion:**

A high prevalence of *T. globulosa* was found in camels in Beheira Governorate, Egypt. This is the first report to confirm the identification of T. globulosa from camel based on morphometrical studies and molecular and phylogenetic analysis in Egypt. More thorough studies on the incidence, molecular, and genetic analysis of *Trichuris* spp. in Egypt are required in addition to camel control programs.

## Background

Throughout the world, one-humped camels (*Camelus dromedarius*) serve various purposes. Many parts of the world use camels as a source of milk, meat, and transportation [1, 2].

Due to improper hygiene practices, several parasites could infect camels as gastro-intestinal nematodes, especially *Trichuris* [3].

Infected camels with parasites produced less milk and meat, had poorer fertility, and had lower calving rates [4]. Camels play a significant role in the epidemiology of parasitic diseases [5]. In Egypt, the camel population was estimated at 80,000, most of which was in upper Egypt.

Helminths that could infect camels include trematodes, cestodes, and nematodes. Whipworms are soil-transmitted nematodes distributed worldwide, parasitizing several mammalian hosts such as ruminants, primates, and rodents [6, 7]. *Trichuris* spp. is one of the most common intestinal parasites affecting both humans and animals, and it can spread directly through the fecal-oral route, resulting in severe illness and financial loss [8, 9].

*Trichuris* is closely related to *Trichinella* and *Capillaria* and belongs to the class Enoplea, subclass Dorylaimia, and order Trichinellida [10]. The diagnosis of *Trichuris* is made by observing eggs in fecal samples in living animals [11].

As many characters used to distinguish between species interfere, *Trichuris* species morphological identification is difficult [12]. The spicule length is the most essential character to differentiate *Trichuris* spp [13].

In the distal portion of the spicule sheath of *T. globulosa*, males exhibit a spherical bulge covered with longer spines than the rest of the sheath. Less than 4.9 mm in spicule length and no prominent vulva in females [13, 14].

While molecular identification has been used successfully, especially when combined with morphometrical analysis [15–18].

Until now, there have been few studies on T. globulosa in camels, especially in Egypt. So, this work aims to identify the prevalence of *T. globulosa* in camels in Beheira Governorate, Egypt, through morphometrical studies, molecular analysis, and phylogenetic analysis.

## Methods

### Study area

**Fig. 1:**
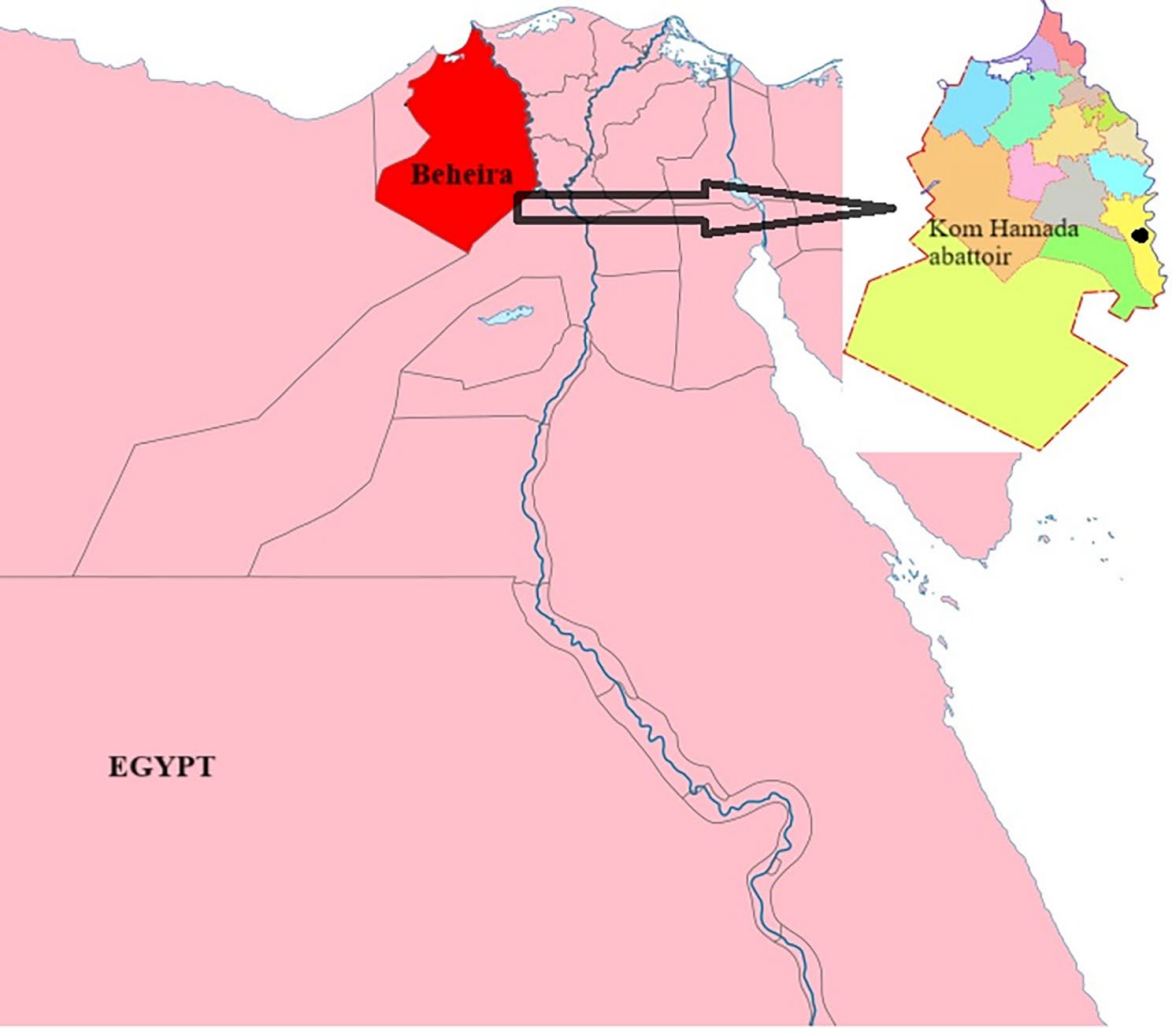
Map of Egypt, illustrating the sampling site

During the period that extended between August 2022 and July 2023, cecum was collected from 127 imported camels aged < 3 years, 3–5 years, and > 5 years, and their numbers were (10, 92 and 25), respectively, at Kom Hamada abattoir in Kom Hamada city, which is located at a latitude of 30°45’45"N and a longitude of 30°41’50"E, Beheira Governorate, Egypt (Fig. 1).

### Samples

Each cecum was ligated and removed from the camels slaughtered at the abattoir, collected in clean bags labeled (age, seasonal dynamics), and transferred to the laboratory of Parasitology in an ice container for further examinations. Initial examination was done within hours (24 h.) after transportation.

### Parasitic materials

In the laboratory of the parasitology Department, Faculty of Veterinary Medicine, Kafrelsheikh University, Egypt, each cecum was opened; contents were evacuated and scrutinized for whipworms with the aid of a hand magnification lens and OLYMPUS stereomicroscope. The worms were collected from the collected cecum. The worms were preserved in formalin 10% and ethyl 70% alcohol for morphometrical identification and molecular analysis.

### Morphological and biometrical examination

After initial morphological examination of collected whipworms, adults (30) of *Trichuris* spp. (15 males and 15 females) represented 15 infected camels were measured using an OLYMPUS microscope equipped with a camera (OLYMPUS DP 28) according to parameters cited by other studies [13–15]. The worms were identified using available keys and descriptions based on morphological features [14, 15, 18–20].

### Molecular examination

Genomic DNA was extracted from three adult *Trichuris* spp. samples using the QIAamp DNA Mini Kit (Catalogue no. 51,304) following the instructions of the manufacturing.

### Molecular characterization

The genes for cytochrome b (Ctyb) and Nuclear small subunit ribosomal RNA (18s rRNA) were subjected to amplification. Fragments of genes amplified using specific primers.

(5 ´-GAGTAATTTTTATAATACGAGAAGT-3 ´) and (5 ´-AATTTTCAGGGTCTCTGCTTCAATA-3 ´) for Cytb as forward and reverse primers, respectively [11].

(5 ´-CGCGAATRGCTCATTACAACAGC-3 ´) and (5 ´- GGGCGGTATCTGATCGCC − 3 ´) for 18 S, as forward and reverse primers, respectively [21].

Cycling conditions and PCR Mix were applied as Callejón et al. [15] and Floyd et al. [21].

Electrophoresis with 1.5% Agarose gel in TAE buffer and stained with 0.5 µg/ml ethidium bromide (Sigma) was used for visualization of PCR products. A 100-bp ladder and, a known sample (positive control), and distilled water (negative control) were included in the gel. A gel documentation system (SYNGENE) was used to take images of the gels, and computer software was used to analyze the data.

### Sequencing and phylogenetic study

Using an Applied Biosystems 3130 automated DNA sequencer (ABI, 3130, USA), a purified PCR product of the Cytb and 18s genes was sequenced forward and backward. Using a ready reaction Bigdye Terminator V3.1 cycle sequencing kit. (Perkin-Elmer/Applied Biosystems, Foster City, CA).

A BLAST analysis (Basic Local Alignment Search Tool) was initially performed to establish sequence identity to GenBank accessions [22]. Higher identity sequences were downloaded from Genbank, and *Trichinella spiralis* was used as an out-group species for phylogenesis. Sequences were aligned using the alignment tool in Mega 11 software [23].

A phylogenetic tree was built using the Maximum Likelihood method and the Tamura Nei model [24].

Finally, the sequences obtained in this work were submitted to Genbank for accession numbers.

### Statistical analysis

A statistical application called GraphPad Prism 9 was used to examine statistical significance differences using a Chi-square. *P* values less than 0.05 were used to determine statistical significance.

## Results

### Survey finding

A total of 35 dromedaries out of 127 investigated had *Trichuris* spp. infection, meaning that the overall prevalence was 27.56%.

The age of the camel affected the infection rate, older animals (> 5 years) having a higher prevalence of infection (24%) than animals of ages (< 3 years) (20%) than animals of ages (3–5 years) (19.14%). However, the statistical analysis indicated these changes were not statistically significant.

According to season: *Trichuris* spp. showed a unique pattern in camels in different seasons: summer (31.25%) > autumn (28.13%) > spring (25.8%) > winter (25%) indicating year-round infection. However, the statistical analysis indicated these changes were not statistically significant.

### Morphological and biometrical results (Fig. 2)

We studied the morphology of thirty adult camel worms (fifteen male and fifteen female) isolated from the camel’s cecum.

The vaginal length of females varied from 0.7 to 0.9 mm with an average: 0.79 mm, while their overall length varied from 37.7 to 59.9 mm (average: 52.9 mm). A morphological analysis revealed that the vulva had a round margin free from spines. Vulva looked smooth. A tiny egg chamber was located anterior to the vulva, and the vagina was short and occasionally slightly convoluted in multiple loops.

Males had a total length of 43.5 to 62.8 mm (average: 53.3 mm), while the spicule length ranged from 3.91 to 4.9 mm (average: 4.4 mm). Spicule diameters varied from 0.031 to 0.075 mm (average of 0.046 mm). The distal portion of the spicule sheath in males featured a spherical bulge covered in spines that were longer than the remaining spines in the sheath.

**Fig. 2:**
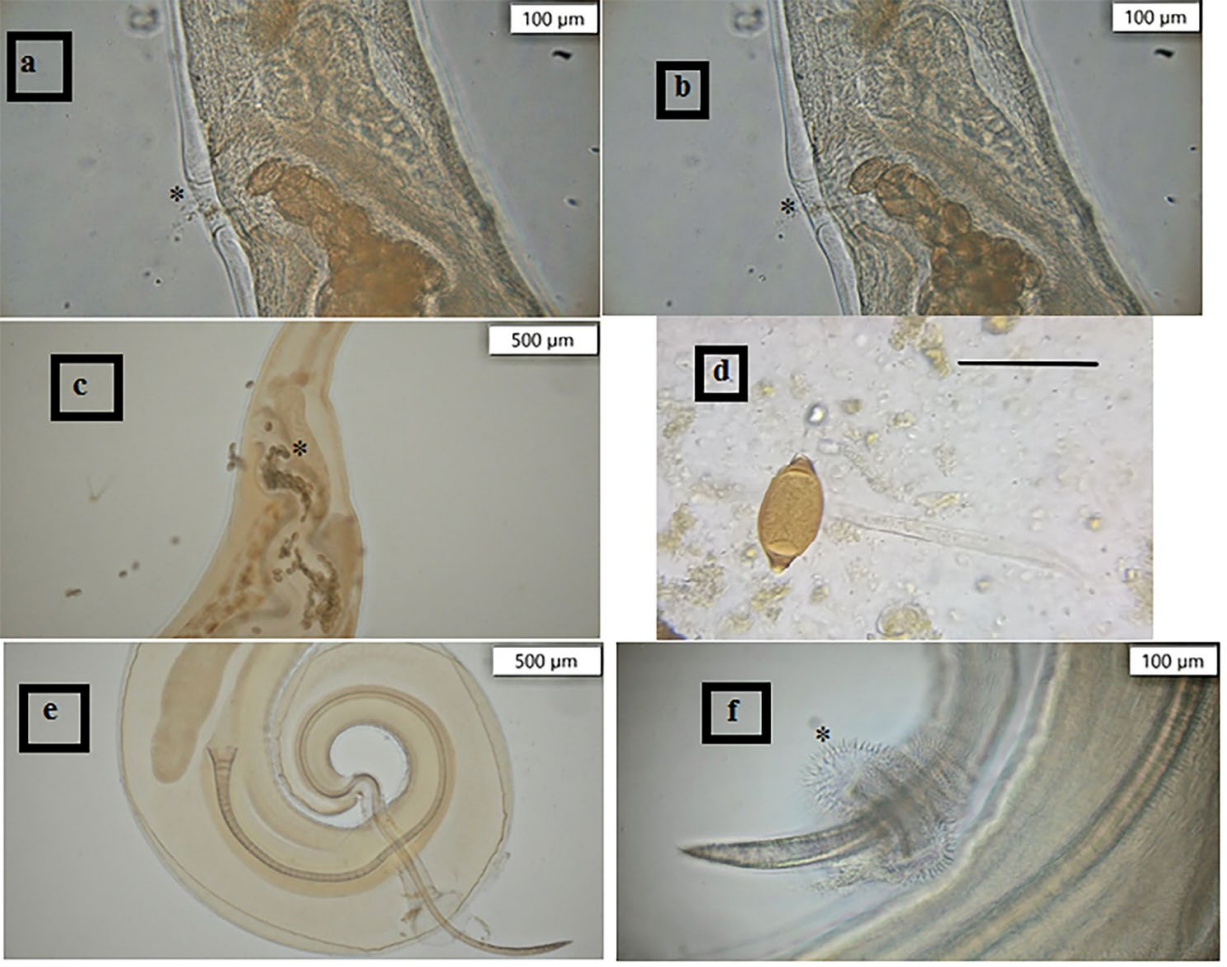
*Trichuris globulosa* detected in the current study: **(a)** and **(b)** female vulva and vagina, **(c)** and **(d)** egg (scale bar 70 μm), **(e)** male caudal region showing the spicule, the evaginated spicule sheath and **(f)** spherical bulge covered with spines

### Molecular results

Using particular primers, the cytb and18s genes were amplified by PCR to produce bands measuring approximately 450 bp and 900 bp, respectively. The adult *T. globulosa* Ctyb sequences were placed in Genbank with accession numbers OR863681, OR863682, and OR863683 and varied between 439 and 444 bp. while the adult *T. globulosa* 18s sequences placed in Genbank with accession numbers OR775092, OR775093, and OR775096 and varied from 786 to 860 bp. The Genbank database was used for the BLAST analysis of the sequenced data, which indicated the existence of the *T. globulosa* isolate from camels based on the 18s and Cytb genes.

**Fig. 3:**
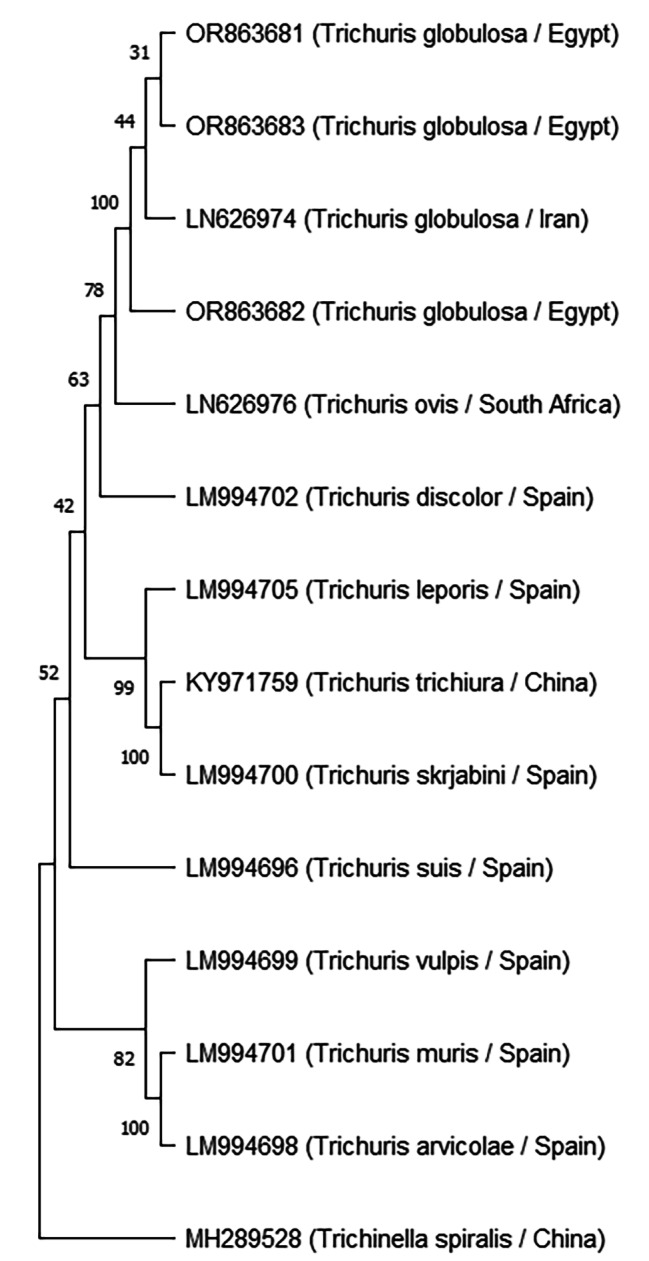
Maximum Likelihood tree based on Cytb DNA gene of *Trichuris globulosa* in the current study, *Trichinella spiralis* was used as the outgroup. * Current study

**Fig. 4:**
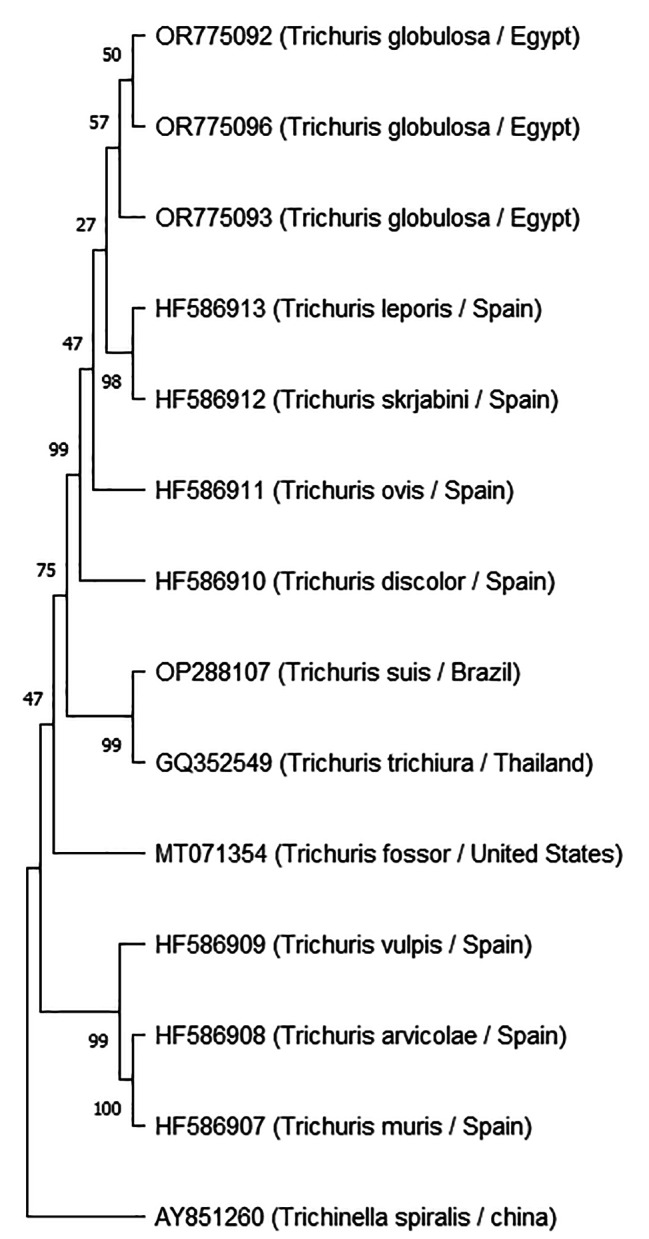
Maximum Likelihood tree based on 18s partial gene of ribosomal RNA gene of *Trichuris globulosa* in the current study, *Trichinella spiralis* was used as the outgroup. * Current study

The Cytb sequences (OR863681, OR863682, and OR863683) had 100% homology to *T. globulosa* recovered from *Camelus dromedaries* in Iran (LN626974) (Fig. 3). While The 18s sequences (OR775092 and OR775093) had 99.88% homology to *T. ovis* recovered from *Capra hircus* in Spain (HF586911) and showed 99.65, 99.62% homology to *T. discolor* recovered from *Bos Taurus* in Spain (HF586910). While OR775096 had 97.2% homology to *T. ovis* recovered from *Capra hircus* in Spain (HF586911), and *T. discolor* recovered from *Bos Taurus* in Spain (HF586910) (Fig. 4).

This study identified *Trichuris* from Egypt as *T. globulosa* based on morphological and molecular examinations.

## Discussion

Important camelid parasites, whipworms (*Trichuris* spp.) are under-estimated parasite infections compared with other gastrointestinal nematodes, although they could result in a significant loss. The large intestine and caecum contain adult parasites. Significant enteritis brought on by *Trichuris* spp. causes diarrhea, dehydration, and weight loss [25]. The most common and widespread trichurid in camels is *Trichuris globulosa* [26].

According to the current study, *Trichuris* spp. was present in 27.56% of the camels in Beheira that had been slaughtered. The findings of the current study were lower than 40% in Iran [27] and higher than the1.5%, 4.1%, 4.9%and 12.24% in Pakistan [28], Iran [29, 30], and Toukh, Egypt [31], respectively. Variations in ecological location, conditions of the environment, animal rearing practices, degree of exposure to infectious eggs, and hygienic system could all contribute to the variation in prevalence.

In this study, *Trichuris globulosa* was detected at all ages, and the infection rates at ages of (< 3 years), (3–5 years) and (> 5 years) were 20%, 19.14%, and 24%. These results were matched those of the previous study [31]. This could be because the older animals have contact with the eggs of *Trichuris* spp. more than young animals.

Summer and spring had the highest infection rates, followed by autumn and then winter. Some studies showed the infection rate was the highest in winter [31]. Differences in environmental conditions, pastures among seasons, and geographical distribution could cause these variations.

There were different species of *Trichuris* affecting camels: *T. tenuis* [32], *T. globulosa* [15], *T. skrjabini* [30], and *T. ovis* [33].

In the present study, the worms were identified as T.globulosa parasitizing *Camelus dromedarius* from Egypt. Similar to females of Trichuris tenuis, T. discolor, and T. globulosa, females of Trichuris spp. of Egyptian camels did not have an everted vagina without spines [13, 15, 34, 35]. In contrast, female T. ovis and *T. skrjabini* were distinguished from each other based on the presence of everted vagina covered in sharp, acute spines (*T. skrjabini*) and large, papilla-like spines (*T. ovis*) [15, 34, 35].

The structure and lining of the vagina and the distance between the vulva and the uterine sphincter are the main factors that determine how differentiable the females of *Trichuris spp*. are from one another. The distance between the vulva and uterine sphincter of the females of *T. globulosa* of Iranian camels varied from 0.7 to 0.8 mm (average: 0.73 mm) [15] in contrast, it varied from 0.7 to 0.9 mm (average: 0.79 mm) in the current study. For *T. discolor*, this distance varied from 3.6 to 5.3 mm [34] and 1.40 to 2.85 mm [36], while *T. tenuis* varied from 2.5 to 3.4 mm [36].

The spicule length was considered the primary characteristic distinguishing *Trichuris* species [13, 15].

The *Trichuris* species key is based on spicule length longer than 5 mm (*T. ovis* = 5.69; *T. tenuis* = 7.2) [35, 37] or spicule length shorter than 5 mm (*T. discolor*) [34, 35].

In line with *T. globulosa*, the male *Trichuris* in the current study on Egyptian camels displayed a range of 4.1 mm to 5.1 mm [13–15, 38] but slightly overlapping *T. ovis*.

The distal end of the spicule of males of *Trichuris* from Egypt is pointed, resembling *T. globulosa*, whereas the distal end of the spicule in *T. tenuis* and *T. discolor* is bluntly rounded [13–15, 35].

Although there are some notable differences between *T. globulosa* and *T. ovis*, these two species are remarkably alike in size and external body shape as well as the size and shape of the eggs (the spicule lengths of 2 species overlapped slightly) [13]. Specifically, the males of both species have a spherical bulge at the distal end of the spicule sheath when fully protruded. *T. globulosa* exhibits noticeably longer protrusion spines, while the protrusion’s spines in *T. ovis* are smaller than those on the rest of the sheath. Overall, spicule sheath spines are generally longer and closer together in *T. globulosa* than those in *T. ovis* [14, 39, 40]. Tenora et al. [38] disagreed with these studies and concluded that there isn’t a difference in character between the two species.

The nuclear small subunit ribosomal RNA (18s rRNA) gene has been widely employed in nematode phylogenetics. This gene was sequenced and used in phylogenetics in more than 1000 species of nematodes [16, 17].

According to the mitochondrial data, there is a different genetic lineage between *T. ovis* and *T. globulosa*. A distinct genetic lineage of *T. ovis* from South African sheep, which would be closely linked to the *T. globulosa* populations found in Iranian camels, was confirmed by cytochrome b partial gene sequences (Ctyb). For the first time, the cytb partial gene sequences of *T. globulosa* have been published [15].

Through BLAST, a comparison of the newly obtained Cytb and 18s sequence with other *Trichuris* sequences on Genbank, the current study’s sequence indicated that the species was *T. globulosa*.

A phylogenetic tree based on Cytb revealed high similarity between *Trichuris globulosa* in the present study and *T. globulosa* from camels in Iran [15], which reported *T. globulosa*’s Cytb partial gene sequences for the first time, and this study reported *T. globulosa*’s 18s partial gene sequences for the first time.

## Conclusion

A high prevalence of *T. globulosa* was found in camels in Beheira Governorate, Egypt. This is the first report to confirm the identification of T. globulosa from camel based on morphometrical studies and molecular and phylogenetic analysis in Egypt. More thorough studies on the incidence, molecular, and genetic analysis of *Trichuris* spp. in Egypt are required in addition to camel control programs.

**Table 1:** *Trichuris globulosa* measurements (mm) from current study with comparison with previous studies

Male	Current study, camel and Egypt	Skrjabin et al., (1957) [13]	Cutillas et al. (1995), copra hircus and Spain [14]	Callejón et al., (2015), camel and Iran [15]
Total length	53.3 (43.5–62.8)	ND	59 (44–74)	ND
Length of anterior part	36.87 (30.35–43.2)	ND	17.5 (12–23)	ND
Length of posterior part	16.48 (12.9–20)	ND	45 (30–60)	ND
Diameter of anterior part	0.188 (0.143–0.225)	ND	ND	0.13 (0.11–0.15)
Diameter of posterior part	1.052 (0.425–1.225)	0.71	ND	0.65 (0.52–0.8)
Length of spicule	4.4 (3.91–4.9)	4.75 (3.8–5.7)	4.69 (4.48–4.9	4.5 (4.1–5.1)
Diameter of spicule	0.046 (0.031–0.075)	0.04 (0.032–0.05)	ND	0.04 (0.03–0.05)
Length of spicule sheath	0.62 (0.44–0.82)	ND	ND	0.62 (0.39–0.78)
Width of spicule sheath	0.165 (0.102–0.296)	ND	ND	0.18 (0.09–0.28)
**Female**				
Total length	52.9 (37.7–59.9)	ND	ND	36 (31–40)
Length of anterior part	40.04 (28.83–50.5)	ND	ND	37 (23–32)
Length of posterior part	10.6 (8.08–13.98)	ND	ND	8 (7–8)
Diameter of anterior part	0.158 (0.133–0.194)	ND	ND	0.14 (0.11–0.16)
Diameter of posterior part	0.95 (0.78–1.23)	0.87	ND	0.73 (0.64–0.82)
Width of uterus	0.76 (0.63–0.9)	ND	ND	ND
Distance from vulva to sphincter of uterus	0.79 (0.7–0.9)	ND	ND	0.73 (0.7–0.8)
Egg length	0.067 (0.065–0.07)	0.06	ND	0.06 (0.06–0.07)
Egg width	0.03 (0.027–0.0325)	0.04	ND	0.03 (0.03–0.04)

ND Not determined

## Data Availability

All data regarding to this manuscript is included in it. Sequences generated were deposited in GenBank under acccesion numbers OR863681, OR863682, OR863683,OR775092, OR775093, and OR775096.

## Abbreviations

Mm: millimeter
18s: the nuclear small subunit ribosomal RNA
Cytb: Cytochrome b
Bp: base pair

## References

[1] Anwar M, Hayat CS (1998). Gastrointestinal parasitic fauna of camel (Camelus dromedarius) slaughtered at Faisalabad abattoir. Pak J Biol Sci.

[2] Mohanapriya T, Saravanan S, Ramprabhu R (2020). Concomitant infection of *Trichuris Globulosa* and *Trichostrongylus* spp. in a dromedary camel. J Entomol Zool Stud.

[3] Parsani HR, Singh V, Momin RR (2008). Common parasitic diseases of camel. Vet World.

[4] Sazmand A, Joachim A. Parasitic diseases of camels in Iran (1931–2017)–a literature review. Parasite. 2017; 24. 10.1051/parasite/2017024 PMC5479402 28617666

[5] Rivero J, Cutillas C, Callejón R (2023). New genetic lineage of whipworm present in bactrian camel (Camelus bactrianus). Vet Parasitol.

[6] Anderson RC. Nematode parasites of vertebrates: their development and transmission. Wallingford, Oxon OX10 8DE, UK: CABI. 2000; 2.

[7] Di Filippo MM, Berrilli F, De Liberato C, Di Giovanni V, D’Amelio S, Friedrich KG, Cavallero S (2020). Molecular characterization of *Trichuris* spp. from captive animals based on mitochondrial markers. Parasitol Int.

[8] Roepstorff A, Mejer H, Nejsum P, Thamsborg SM (2011). Helminth parasites in pigs: new challenges in pig production and current research highlights. Vet Parasitol.

[9] Jex AR, Nejsum P, Schwarz EM, Hu L, Young ND, Hall RS, Korhonen PK, Liao S, Thamsborg S, Xia J, Xu P, Waang S, Scheerlinck JP, Hofmann A, Sternberg PW, Wang J, Gasser RB (2014). Genome and transcriptome of the porcine whipworm *Trichuris Suis*. Nat Genet.

[10] Liu GH, Wang Y, Xu MJ, Zhou DH, Ye YG, Li JY, Song HQ, Lin RQ, Zhu XQ. Characterization of the complete mitochondrial genomes of two whipworms *Trichuris ovis* and *Trichuris discolor* (Nematoda: Trichuridae). Infect Genet Evolut. 2012; 12(8):1635–1641. 10.1016/j.meegid.2012.08.005 22926017

[11] Rivero J, Zurita A, Cutillas C, Callejón R (2022). The use of MALDI-TOF MS as a diagnostic tool for adult *Trichuris* species. Front Vet Sci.

[12] Gagarin VG. Critical evaluation of measured characters in the differentiation of helminth species (on the model of trichocephalids). Fauna gelmintov zivotnych y rastenij kirgizii. 1974; 3–9.

[13] Skrjabin KI, Shikhobalova NP, Orlow IV. Trichocephalidae and Capillariidae of animals and the man and the diseases caused by them. In: Birron, A., Greenberg, D, editors, Essentials of Nematodology. Israel Program for Scientific Translations Ltd. Keter Press Wiener Binder Ltd., Jerusalem, vol. VI; 1957.

[14] Cutillas C, German P, Arias P, Guevara D (1995). *Trichuris ovis* and *Trichuris globulosa*: morphological, biometrical, and genetic studies. Exp Parasitol.

[15] Callejón R, Gutiérrez-Avilés L, Halajian A, Zurita A, de Rojas M, Cutillas C (2015). Taxonomy and phylogeny of *Trichuris Globulosa* Von Linstow, 1901 from camels. A review of *Trichuris* species parasitizing herbivorous. Infect Genet Evol.

[16] Blaxter ML, De Ley P, Garey JR, Liu LX, Scheldeman P, Vierstraete A, Vanfleteren JR, Mackey YL, Dorris M, Frisse LM, Vida JT (1998). Thomas, WK. A molecular evolutionary framework for the phylum Nematoda. Nat.

[17] Callejón R, Nadler S, De Rojas M, Zurita A, Petrášová J, Cutillas C (2013). Molecular characterization and phylogeny of whipworm nematodes inferred from DNA sequences of cox1 mtDNA and 18s rRNA. Parasitol Res.

[18] Oliveros R, Cutillas C, De Rojas M, Arias P (2000). Characterization of four species of *Trichuris* (Nematoda: Enoplida) by their second internal transcribed spacer ribosomal DNA sequence. Parasitol Res.

[19] Yamaguti S (1961). Systema Helminthum. Volume III. The nematodes of vertebrates.

[20] Soulsby EJL. Helminths. Arthropods and Protozoa of domesticated animals. 1982; pp, 291.

[21] Floyd RM, Rogers AD, Lambshead PJD, Smith CR (2005). Nematode-specific PCR primers for the 18S small subunit rRNA gene. Mol Ecol.

[22] Altschul SF, Gish W, Miller W, Myers EW, Lipman DJ (1990). Basic local alignment search tool. J mol boil.

[23] Tamura K, Stecher G, Kumar S (2021). MEGA11: molecular evolutionary genetics analysis version 11. Mol biol evol.

[24] Tamura K, Nei M (1993). Estimation of the number of nucleotide substitutions in the control region of mitochondrial DNA in humans and chimpanzees. Mol Boil Evol.

[25] Fowler (1996). Husbandry and diseases of camelids. Revue Scientifique Et Technique (International Office Epizootics).

[26] Dakkak A, Ouhelli H. Helminths and helminthoses of the dromedary. A review of the literature. Revue Scientifique et Technique de l’OIE (France); 1987. 10.20506/rst.6.2.302 32370332

[27] Borji H, Razmi GR, Movasaghi A, Naghibi AA, Maleki M (2010). A study on gastrointestinal helminths of camels in Mashhad abattoir. Iran Iran J Vet Res.

[28] Hayat CS, Hayat B, Maqbool A, Badar N, Hashmi HA, Hussain I (1998). Common gastrointestinal helminths of camels of Pakistan. J Camel Pract Res.

[29] Anwar AH, Khan MN. Parasitic fauna of camel in Pakistan. In Proceedings of the Third Annual Meeting for Animal Production under Arid Conditions Vol. 2. 1998; pp. 69–76.

[30] Anvari-Tafti M, Sazmand A, Hekmatimoghaddam S, Moobedi I (2013). Gastrointestinal helminths of camels (Camelus dromedarius) in center of Iran. Trop Biomed.

[31] Ahmed NE, El-Akabway LM, Ramadan MY, El-Gawad SMA (2013). Detection and identification of some helminth parasites affecting camels. Egypt J Vet Sci.

[32] Chandler AC (1930). Specific characters in the genus *Trichuris*, with a description of a new species, *Trichuris Tenuis*, from a camel. J Parasitol.

[33] El Bihari S. 5. Helminths of the camel: A review. Br Vet J. 1985; 141(3):315–326. 10.1016/0007-1935(85)90070-3 3891009

[34] Knight (1971). Redescription of Trichuris discolor (Von Linstow, 1906) and T. Skrjabini (Baskakov, 1924) from domestic ruminants in the United States and comparisons with T. ovis (Abilfgaar, 1795). J Parasitol.

[35] Rickard LG, Bishop JK (1991). Redescription of *Trichuris tenuis* Chandler, 1930, from llamas (Lama glama) in Oregon with a key to the species of Trichuris present in north American ruminants. J Parasitol.

[36] Callejón R, Halajian A, de Rojas M, Marrugal A, Guevara DC, Cutillas C (2012). 16S partial gene DNA and internal transcribes spacers ribosomal DNA as differential markers of *Trichuris discolor* populations. Vet Parasitol.

[37] Oliveros R, Cutillas C (2003). Redescripción De Trichuris ovis (Nematoda) (Abildgaard, 1795) parásito de *Ovis aries* (Linné, 1758) y *Capra hircus* (Linné, 1758). Rev Iber Parasitol.

[38] Tenora F, Barus V, Spakulova M, Cutillas C (1997). Scanning electron microscopy on males of two *trichuris* (Nematoda) species parasitizing the hosts ovis and capra. Acta Univ Agric.

[39] Sprehn EW. Lehrbuch der Helminthologie, Berlin; 1927.

[40] Baylis HA (1932). Three notes on parasitic nematodes. Ann Mus Nat Hist.

